# Enhancement of Quality and Safety of Low-Salt *Pixian* Douban Fermentation with *Paenibacillus polymyxa* M17 27-6

**DOI:** 10.3390/foods14244200

**Published:** 2025-12-07

**Authors:** Zirong Gao, Weihong Tao, Xiaolei Ren, Ningbo Qin, Yingxi Chen, Chaofan Ji, Xinping Lin, Yiwei Dai, Sufang Zhang

**Affiliations:** SKL of Marine Food Processing & Safety Control, National Engineering Research Center of Seafood, Collaborative Innovation Center of Seafood Deep Processing, School of Food Science and Technology, Dalian Polytechnic University, Dalian 116034, China; zrgao1015@163.com (Z.G.); twh12345600@163.com (W.T.); 18330261623@163.com (X.R.); qinningbo6902765@163.com (N.Q.); yingxichen24@163.com (Y.C.); jichaofan@outlook.com (C.J.); yingchaer@163.com (X.L.)

**Keywords:** *pixian* douban, fermentation, low-salt, *Paenibacillus polymyxa* M17 27-6, volatile compounds

## Abstract

Traditional *pixian* douban is characterized by elevated salt concentrations, often exceeding 12%. Given the established correlation between high-salt diets and various health disorders, the necessity for effective salinity reduction becomes evident. However, the reduction in salt content may result in quality deterioration. To address the adverse effects associated with decreased salt concentration, the strain *Paenibacillus polymyxa* M17 27-6, which possesses the capability to produce antimicrobial compounds, was used in the fermentation of low-salt *pixian* douban. Additionally, we employed low-salt uninoculated and high-salt uninoculated groups as fermentation controls, with the entire fermentation cycle lasting 35 d. In terms of safety, microbial diversity sequencing and the content of biogenic amines and aflatoxin B1 were conducted. Microbial diversity sequencing analyses indicated the presence of potentially pathogenic *Escherichia* and *Shigella*, as well as the spoilage-causing *Trichosporon* and *Issatchenkia*, in the uninoculated low-salt group, whereas no contaminating bacteria were detected in the inoculated group. Relative to the uninoculated low-salt group, levels of aflatoxin B1 and biogenic amines were significantly reduced. In terms of quality and volatiles, compared to the uninoculated high-salt group, concentrations of amino acid nitrogen and total acids increased to 0.93 g/100 g and 1.21 g/100 g, respectively, alongside significantly enhanced levels of organic acids and antioxidant activity. At the same time, volatile compound content and abundance increased. In conclusion, the incorporation of *P. polymyxa* M17 27-6 in the production of low-salt broad-bean paste effectively enhances quality and safety and provides a theoretical basis for developing low-salt *pixian* douban products.

## 1. Introduction

Traditional bean-fermented foods, with a history spanning millennia, serve as popular condiments, such as broad-bean paste, soy sauce, natto, and curd [1]. Regional variations in raw materials and production methods contribute to the distinct flavor profiles of bean-fermented foods [2]. Broad-bean paste has attracted attention not only as a nutritious source of amino acids, minerals and vitamins [3], but also for its health benefits, which include diverse functional properties such as antioxidant, anti-inflammatory, fibrinolytic, anti-obesity, and anticancer activities [4].

Traditionally, broad-bean paste contains a salt concentration of 12% or more [5]. While high salinity can inhibit harmful microorganisms, it also poses health risks, including hypertension and diabetes [6]. Excessive salt levels can suppress beneficial microorganisms, detrimentally affecting the quality of *pixian* douban. Recent initiatives to reduce dietary salt intake have gained attention. Moreover, many countries have already implemented comprehensive salt reduction policies. For instance, China’s National Nutrition Plan (2017–2030) proposes reducing per capita sodium intake by 20% by 2030 [7]. However, simply reducing the salt content in fermented products could increase safety risks and alter sensory attributes. Seo et al. found that reduced salt levels in broad-bean paste fermentation promoted the growth of harmful microorganisms like *Klebsiella*, *Cronobacter*, and *Acinetobacter* [8].

Low-salt fermentation causes changes in the microbial composition, which affects the flavor and safety of the product [9]. The reduction in salt can alter enzymatic activities, leading to changes in the production of flavor compounds [10]. While some studies have focused on the influence of functional strains on flavor profiles, the exact mechanisms through which these microorganisms influence the sensory properties of low-salt fermented foods are still unclear [11]. Moreover, flavor formation in low-salt conditions is often insufficient to match the complexity of high-salt products, which can be detrimental to consumer acceptance. Biogenic amines, such as histamine and tyramine, are naturally formed during fermentation through the decarboxylation of amino acids by certain microorganisms [12]. The reduction in salt content can exacerbate the formation of biogenic amines due to the proliferation of specific bacteria, which pose health risks such as headaches, high blood pressure, and food poisoning. Although the use of certain microbial strains has been shown to reduce biogenic amine formation, much remains to be understood regarding the balance between salt concentration, microbial activity, and the production of these compounds. Traditional methods of inoculating microorganisms are not enough to ensure high-quality production, so we need to introduce beneficial microorganisms that can prevent harmful microbes and reduce undesirable compounds [13]. Devanthi et al. enhanced the flavor of low-salt soy sauce (6% salt) through the inoculation of *Zygosaccharomyces rouxii* and *Tetragenococcus halophilus* [14]. Using *Pichia fermentans*, *Kodamaea ohmeri*, and *Lactococcus lactis* with α-ketoglutaric acid improved the quality of low-salt bean paste by enhancing enzyme activities linked to flavor development [15]. The strains *Torulaspora delbrueckii* and *Pichia guilliermondii* were used in soy sauce fermentation, resulting in elevated production of ethanol and 3-methyl-1-butanol while inhibiting undesirable bacteria [16]. Zhao et al. found a significant correlation between volatile components and LAB, suggesting that LAB influences the aroma of doubanjiang [17]. Previous studies indicated that the primary functional bacteria in the fermentation of *pixian* douban predominantly include *lactic acid* bacteria, *Bacillus* species, and *Zygosaccharomyces rouxii*. In low-salt environments, the proliferation of spoilage and pathogenic bacteria often suppresses the metabolic activity of beneficial microorganisms, slowing fermentation rates and hindering the formation of flavor compounds [18]. Certain beneficial strains—such as lactic acid bacteria and Saccharomyces cerevisiae—cannot tolerate salinity exceeding 6% without evolution and genetic engineering. In contrast, the selected *P. polymyxa* M17 27-6 strain can grow normally at 15% salinity. Therefore, screening strains with antibacterial properties, good safety, and multifunctional capabilities, and investigating their impact on the quality and safety of low-salt fermented *pixian* douban is crucial.

*Bacillus* species are key bacteria in broad-bean paste fermentation, playing a major role in shaping the product’s quality and flavor [19]. Most *Bacillus* species are considered safe for consumption and are commonly used in the food industry. *Bacillus* can produce antimicrobial peptides, enzymes, and volatile organic compounds that inhibit foodborne pathogens and spoilage organisms, showing potential in microbial contamination control [20]. Studies have shown that *Bacillus* subtilis is more effective than lactic acid bacteria (LAB) at preventing aflatoxin production. *Bacillus* also produces antimicrobial substances like lipopeptides and proteins and releases enzymes that help break down proteins during fermentation. These proteases facilitate the degradation of proteins into peptides and amino acids, enhancing texture, flavor, and the production of bioactive peptides, while providing precursors for flavor volatiles [21]. Wang et al. found that co-fermentation with *B. subtilis* and *B. licheniformis* in Moutai Baijiu production improved glucose metabolism and flavor compound synthesis [22]. The probiotic strain *B. licheniformis* B4, derived from camel feces, showed phytase, protease, cellulase, and xylanase activities, reducing phytate levels and improving the nutritional quality of soybean meal in solid-state fermentation [23]. Additionally, the *B. thuringiensis* ATCC10792 strain was used to inhibit fungal proliferation and enhance quality during cocoa bean fermentation [24]. *Paenibacillus polymyxa* has also been shown to produce antibiotics and exhibit antagonistic effects against *Fusarium* [25]. Meanwhile, studies have also indicated that *Paenibacillus polymyxa* is a promising antagonistic bacterial strain capable of effectively suppressing *Pseudomonas syringae* [26].

Based on our previous experiments, a strain named *Paenibacillus polymyxa* M17 27-6 that exhibits antibacterial activity, enzyme production capability, and excellent safety and fermentation performance was screened (experimental data not yet published). In this study, the strain *P. polymyxa* M17 27-6 was employed to mitigate harmful bacteria. This strain was applied in the fermentation of low-salt *pixian* douban to evaluate its impact on quality, aiming to achieve regulatory control in traditional *pixian* douban production. This approach supports the advancement of low-salt fermented foods and the industrialization of traditional low-salt fermented products.

## 2. Materials and Methods

### 2.1. Materials

The raw materials for the preparation of *pixian* douban included moldy broad beans, chili peppers, and salt, which were sourced from local farmers in *Pixian*, Sichuan. The moldy broad beans were uniform in size, free of discoloration, and classified as Grade A beans. The culture media used in this study were procured from Hopebio Biotechnology Co., Ltd. (Qingdao, China). All chemical reagents are analytical grade and supplied by Tianjin Damao Chemical Reagent Factory (Tianjin, China), Dalian Bonuo Biochemical Co., Ltd. (Dalian, China), and Sigma-Aldrich (St. Louis, MO, USA).

The strain *P. polymyxa* M17 27-6, isolated from *pixian* douban, exhibited significant inhibitory effects against seven common foodborne pathogens (Supplementary Figure S1), including *Listeria monocytogenes* CICC 21635, *Enterococcus faecalis* CICC 23658, *Staphylococcus aureus* CICC 23656, *Escherichia coli* CICC 23657, *Pseudomonas aeruginosa* CICC 21636, *Salmonella enterica* subsp. *Enterica serovar Typhi* CICC 10871, and *Shigella flexneri* CICC 10865. As described in a previously published article, this strain exhibited notable safety and fermentation performance [27]. The strain was inoculated on M17 solid medium and incubated at 37 °C for 24 h. It was identified by 16S rDNA sequencing, stored at −80 °C, and used for producing antimicrobial substances in this study.

### 2.2. Preparation of Pixian Douban

The preparation of *pixian* douban comprises three principal steps: chili embryo fermentation, strain preparation, and *pixian* douban fermentation. Initially, chili peppers were thoroughly cleaned, de-stoned, crushed, and fermented in a 3% (*w*/*v*) brine solution for 72 h. *P. polymyxa* M17 27-6 was streaked onto M17 solid medium and cultured in M17 liquid medium at 200 rpm for 24 h. The liquid-cultured *P. polymyxa* M17 27-6 was then centrifuged at 8000 rpm for 10 min at 4 °C, washed with 0.85% (*w*/*v*) sterile saline, and inoculated into the *pixian* douban, achieving a final concentration of 10^8^ CFU/mL. For the *pixian* douban fermentation, moldy broad-bean and chili embryos were mixed in a 1:1 ratio within a sterile fermenter. Three experimental groups were established with varying salt concentrations: Group A, the low-salt experimental group (6% *w*/*w* salt) inoculated with *P. polymyxa* M17 27-6; Group B, the low-salt control group (6% *w*/*w* salt) without fermentation agent; and Group C, the high-salt control group (15% *w*/*w* salt) without fermentation agent. Fermentation proceeded at room temperature for 35 d. During the first 7 d, the mixture was stirred daily, then every 7 d thereafter. Samples were collected at regular intervals and stored at −80 °C for subsequent analysis.

### 2.3. Determination of Physicochemical Parameters During Fermentation

Basic physicochemical parameters, including salinity content, water content, pH, total acid, and amino acid nitrogen (AAN), were assessed. The water content and AAN were determined according to the national standard of China GB5009.3-2016 [28] and GB5009.235-2016 [29], respectively. The pH value was measured using a pH meter (FE28-Standard, Mettler-Toledo International Trading Co., Ltd., Shanghai, China), while total acid was quantified using the acid-base titration method [30].

### 2.4. Determination of Safety Indicators

#### 2.4.1. Determination of Biogenic Amines

The steps used for the procedure are as follows: Accurately weigh 5.00 g of *pixian* douban sample, add 20 mL of 5% (*w*/*v*) trichloroacetic acid solution, and use a magnetic stirrer for 60 min. Centrifuge and repeat the process twice. Collect the supernatant, dilute to 50 mL, and filter through filter paper. Take 10 mL of the filtrate, add 10 mL of n-hexane, and shake and stir thoroughly. Discard the upper layer and collect the lower layer. Repeat this process twice and set aside for later use. After completing these steps, a 1 mL aliquot of the bioamine extract was alkalized by the addition of 200 μL of 1 mol/L sodium hydroxide solution, followed by buffering with 300 μL of saturated sodium bicarbonate solution. Subsequently, 1 mL of dansulfonyl chloride acetone solution was added, and the mixture was incubated in the dark for 45 min. The reaction was quenched by adding 100 μL of ammonia solution, and the mixture was allowed to stand at room temperature for 30 min to remove residual solvent. The volume was adjusted to 5 mL with acetonitrile, and the sample was centrifuged at 4 °C and 3000 rpm for 5 min. Finally, the processed sample was filtered through a membrane filter prior to analysis and detection.

The derivatization conditions and high-performance liquid chromatography (HPLC) parameters were adapted from Chen et al. [31]. The column was C18 (4.6 mm × 250 mm, 5 µm), with a flow rate of 1 mL/min. The UV detection wavelength was 254 nm, the injection volume was 10 µL, and the column temperature was 30 °C. Mobile phase A was water, and mobile phase B was acetonitrile. The gradient elution program is shown in Supplementary Table S1.

#### 2.4.2. Determination of Aflatoxin B1 and Foodborne Pathogenic Bacteria

The determination of aflatoxin B1 in *pixian* douban was carried out following the instructions provided with aflatoxin B1 enzyme immunoassay kit (YB-9601B1, Huamei Biological Co., Ltd., Wuhan, China). Furthermore, Violet Red Bile Agar (VRBA), Bismuth Sulphite Agar (BS), Baird-Parker Agar, and Salmonella Shigella Agar (SS) were employed to detect foodborne pathogenic bacteria in the various groups of *pixian* douban according to GB4789.2-2022 [32].

### 2.5. Determination of Bioactive Compounds and Antioxidant Activity

#### 2.5.1. Determination of Bioactive Compounds

Total phenol content was quantified by the formaldehyde method, as described by Kim et al. [33]. A volume of 100 μL of the sample was oxidized by the addition of 1 mL of Folin–Ciocalteu’s phenol reagent. The reaction was subsequently neutralized with 20% (*w*/*v*) sodium carbonate solution and allowed to incubate in the dark for 2 h. Gallic acid was used as the standard reference, and the absorbance was measured at 765 nm; the standard curve is y = 0.0886x + 0.0752, R^2^ = 0.9961.

The total flavonoid content was determined by the aluminum chloride colorimetric method, following the protocol outlined by Kim et al. [34]. Rutin was used as the standard, and the absorbance was measured at 510 nm; the standard curve is y = 0.0919x + 0.0557, R^2^ = 0.9971.

#### 2.5.2. Determination of Antioxidant Activity

The antioxidant activity of the fermented *pixian* douban was evaluated through DPPH and ABTS free radical scavenging assays, with modifications based on the methodology of [35].

DPPH free radical scavenging activity: 2 mL of filtered liquid and 2 mL of 100% ethanol DPPH solution (0.2 mM) were mixed in a 10 mL centrifuge tube, (BioCity, Beijing, China) and incubated in the dark at 25 °C for 30 min. Fermented liquid and ethanol alone were used as blanks, while PBS and DPPH ethanol solutions acted as controls. The supernatant was collected after centrifugation at 2330× *g* for 10 min. Absorbance was measured at 517 nm and in triplicate. The scavenging activity was calculated using the following formula:

DPPH free radical scavenging activity (%) = [1 − (Ai-Aj)/Ac] × 100(1)

where Ai, Aj and Ac represent the absorbance values of filtered liquid plus 1 mL of DPPH ethanol solution, filtered liquid plus 1 mL of anhydrous ethanol and PBS plus 1 mL of DPPH ethanol solution, respectively.

ABTS free radical scavenging activity: ABTS (14 mM) and potassium persulfate (5 mM) were dissolved in 0.1 M potassium dihydrogen phosphate buffer (pH 7.4), mixed in a 1:1 ratio, and reacted for 12–16 h at 25 °C. 900 μL of ABTS solution was added to 100 μL of *pixian* douban filtered liquid and incubated in the dark at 25 °C for 15 min. After centrifugation at 14,000× *g* for 1 min, the supernatant was collected, and absorbance was measured at 734 nm in triplicate. The scavenging activity was calculated using the following formula:

ABTS free radical scavenging activity (%) = (1 − As/Ac) × 100(2)

where As and Ac represent the absorbance values of 100 μL of *pixian* douban filtered liquid plus 900 μL of ABTS solution and 1 mL of ABTS solution, respectively.

### 2.6. Determination of Organic Acids

The determination of organic acids in fermented *pixian* douban was referenced from Zhao et al. [36]. The analysis utilized HPLC (Agilent Infinity II, Agilent Technologies Inc., Palo Alto, CA, USA) equipped with a Shim-pack GIST C18-AQ column (4.6 mm × 150 mm × 5 μm, Shimadzu, Kyoto, Japan). The mobile phase A consisted of 0.01 mol/L ammonium dihydrogen phosphate (pH = 2.5), while mobile phase B comprised 95% methanol suitable for HPLC applications. The flow rate was set at 1 mL/min, employing isocratic elution at ambient temperature with the composition of mobile phase A to mobile phase B maintained at 95:5. Organic acids were detected at 210 nm with a UV detector, with each injection volume being 20 µL. The standards used for organic acids included tartaric acid, malic acid, lactic acid, acetic acid, citric acid, and succinic acid, with standard concentrations established at 50, 62.5, 83.3, 125, 250, and 500 ppm.

### 2.7. Determination of Volatile Compounds

#### 2.7.1. Determination of Volatile Compounds by SPME-GC-MS

Volatile compounds in *pixian* douban were analyzed using solid-phase microextraction gas chromatography–mass spectrometry (SPME-GC-MS) (5977B, Agilent, Santa Clara, CA, USA), following the methodology established by Lin et al., with certain modifications [37]. The *pixian* douban samples were placed in a headspace sampling bottle with 50 mg/L cyclohexanone as internal standard. The extraction flask was maintained in a thermostatic water bath at 60 °C and incubated for 25 min. At the conclusion of the incubation period, the tip of the solid-phase microextraction fiber was exposed to the headspace within the injection vial without making contact with the sample. The extraction conditions involved an ambient temperature of 60 °C for 25 min. The fiber was desorbed at a temperature of 250 °C for 5 min, with the aging condition of the extraction head also set at 250 °C for 30 min. Chromatographic conditions were as follows: An Agilent 5977B mass spectrometer was used, equipped with a capillary column (HP-5MS, 30 m × 0.25 mm × 0.25 µm) for separation. The saturated alkanes (C_8_–C_26_) were analyzed under the same gas chromatographic conditions, the retention indices (RI) were calculated, and the compounds were searched and compared using the NIST14 spectral library. An internal standard method was employed for the semi-quantification of volatile flavor compounds by comparing the gas chromatographic peak areas.

#### 2.7.2. Determination of Volatile Compounds by HS-GC-IMS

According to Wei et al., volatile compounds were also analyzed using a headspace–gas chromatography–ion mobility spectrometry (HS-GC-IMS) system (FlavourSpec^®^, G.A.S. mbH, Dortmund, Germany) [38]. An MXT-WAX column (30 m × 0.53 mm × 1 μm; Shimadzu, Kyoto, Japan) was utilized in the system. For each analysis, 2.0 g of the different *pixian* douban samples were accurately weighed into a 2 mL headspace vial and subsequently incubated at 60 °C for 10 min before injection.

### 2.8. Determination of Microbial Diversity

Genomic DNA was extracted from microbial communities and performed using the PowerSoil^®^ DNA isolation kit. Polymerase chain reaction (PCR) amplification of the V3-V4 region of the bacterial 16S rRNA gene and the ITS2 region of the fungal rRNA gene was carried out using two sets of universal primers, 338F (5′-ACTCCTACGGGAGGCAGCA-3′)/806R (5′-GGACTACHVGGGTWTCTAAT-3′) for bacterial amplification and ITS1F (5′-CTTGTCATTTAGAGGAAGTAA-3′)/ITS2R (5′-GCTGCGTTCTTCATCGATGC-3′) for fungal amplification. The resulting PCR products were pooled and subsequently sent to Meiji Biomedical Technology Co., Ltd. (Beijing, China), where sequencing was performed using the Illumina HiSeq 2500 platform.

### 2.9. Determination of Electronic Sensory

#### 2.9.1. Determination of Electronic Nose

Electronic nose analysis was performed using an electronic nose system (PEN3, Airsense, Schwerin, Germany), equipped with ten metal–oxide–semiconductor sensors, the performance characteristics of which are detailed in Supplementary Table S2. For sample pre-treatment, 5.0 g of sample was mixed with 100 mL of water and homogenized. Subsequently, 2 mL of this homogenate was transferred to a headspace flask and incubated in a 60 °C water bath for 20 min before measurements were taken with the electronic nose. The measurement parameters for the electronic nose system are as follows: The sample interval is set to 1.0 s, with a flush time of 12.0 s. The zero-point trim time and presampling time are both set to 5.0 s. The measurement time is configured for 90.0 s. The gas flow is set to 150 mL/min, with the chamber flow and initial injection flow both also set to 150 mL/min.

#### 2.9.2. Determination of Electronic Tongue

Electronic tongue analysis was performed using the TS-5000Z Taste Analysis System (TS-5000Z, Insent, Kanagawa, Japan). For pre-treatment, 5.0 g of sample was homogenized with 100 mL of water and filtered through a 0.22 µm aqueous filter membrane. Five electrodes, namely, sour electrode, salty electrode, fresh electrode, astringent electrode, and bitter electrode, were used to measure the flavor changes in the samples to be tested after fermentation.

### 2.10. Statistical Analysis

SPSS 20.0 software (International Business Machines Corp., Delaware, USA) was used for data statistics, and one-way ANOVA with General Linear Model (GLM) was applied for significant difference analysis. Line and bar graphs were plotted using Origin 2021 software (OriginLab Corp., Massachusetts, USA). Heat maps were plotted using TBtools 2.0 software. HS-GC-IMS data were processed by VOCal software (v0.4.03), GC-IMS Library Search (NIST database and IMS database), and two plugins. All experiments were performed in triplicate, and a *p*-value < 0.05 was considered statistically significant.

## 3. Results

### 3.1. Physicochemical Parameters Analysis of Pixian Douban

The physicochemical parameters of various *pixian* douban formulations were systematically assessed. To enhance clarity, the low-salt experimental group with a 6% salt concentration inoculated with *P. polymyxa* M17 27-6 is designated as “6% + M17 27-6”, the low-salt group without inoculation as “6%”, and the high-salt experimental group with 15% salt concentration as “15%”.

**Figure 1:**
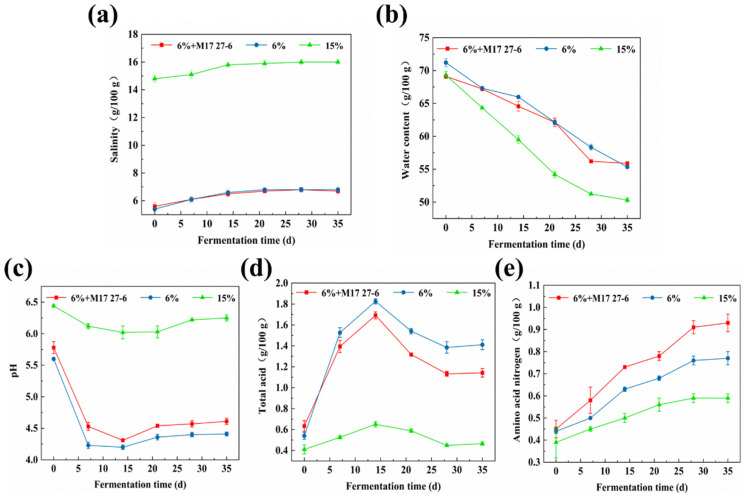
(**a**) Salinity levels of the “6% + M17 27-6”, “6%” and “15%” groups; (**b**) Water content of the “6% + M17 27-6”, “6%” and “15%” groups; (**c**) pH values of the “6% + M17 27-6”, “6%” and “15%” groups; (**d**) Total acid content of the “6% + M17 27-6”, “6%” and “15%” groups; (**e**) Amino acid nitrogen concentrations of the “6% + M17 27-6”, “6%” and “15%” groups.

Salinity changes in different *pixian* douban groups are illustrated in Figure 1a. At the onset of the fermentation, the initial salinity values of “6% + M17 27-6”, “6%” and “15%” groups were approximately 6.13 ± 0.15 g/100 g, 6.13 ± 0.21 g/100 g, and 15.13 ± 0.30 g/100 g, respectively. As fermentation progressed, salinity levels increased, reaching 6.70 ± 0.20 g/100 g, 6.81 ± 0.20 g/100 g, and 15.96 ± 0.18 g/100 g in the respective groups by the late fermentation stage. This increase can be attributed to water evaporation during fermentation, which resulted in a higher salt concentration in the overall fermentation system. Salt plays a crucial role in fermentation, influencing microbial dynamics and creating osmotic pressure that inhibits the growth of harmful microorganisms.

The water content in the *pixian* douban is demonstrated in Figure 1b, revealing a decreasing trend throughout fermentation. The water content is crucial as it significantly impacts microbial growth and metabolism, which in turn influences biochemical reactions and affects the flavor, quality, and nutritional composition of the product. Additionally, the pH values of the three *pixian* douban groups (“6% + M17 27-6”, “6%” and “15%”) decreased from initial values of 5.78 ± 0.10, 5.60 ± 0.02, and 6.44 ± 0.21 to 4.61 ± 0.05, 4.41 ± 0.03, and 6.25 ± 0.05, respectively, by the end of fermentation (Figure 1c). The lower pH in the 6% salt group was attributed to increased lactic acid bacteria (LAB), which produced more organic acid compared to the 15% salt group. The total acid of the three groups (“6% + M17 27-6”, “6%” and “15%”) is shown in Figure 1d, with initial values of 0.63 ± 0.05, 0.54 ± 0.03 and 0.41 ± 0.05 g/100 g, respectively. As fermentation progressed, the total acid content exhibited a trend corresponding to changes in pH. After 35 d of fermentation, the total acid contents of the three groups reached 1.14 ± 0.04, 1.41 ± 0.05, and 0.47 ± 0.01 g/100 g, respectively, all remaining within the acceptable limit of 2.0 g/100 g as specified by the Chinese national standard for *pixian* douban (GB/T 20560-2006 [39]). These findings were consistent with Kim et al., suggesting that appropriate acidity enhances the flavor of pixian Douban [40]. Conversely, excessively high total acid content could lead to the formation of undesirable strong acids that compromise the quality of *pixian* douban [41]. The observed decrease in pH and the higher total acid content (Figure 1d) of low-salt fermented *pixian* douban may be due to reduced inhibition of acid-producing microorganisms, such as *lactobacilli*, which were able to proliferate more easily [42].

Amino acid nitrogen is an important indicator of *pixian* douban quality, with higher concentrations associated with better quality [43]. As shown in Figure 1e, the amino acid nitrogen content of the three groups (“6% + M17 27-6”, “6%”, and “15”) increased continuously during fermentation, reaching 0.93 ± 0.04 g/100 g, 0.77 ± 0.03 g/100 g, and 0.59 ± 0.02 g/100 g, respectively, after 35 d of fermentation. Amino acid nitrogen accumulation peaked in the late fermentation stages, with the increase stabilizing over time [44]. Furthermore, under low-salt conditions, enhanced microbial nitrogen metabolism increased amino acid nitrogen content, thereby improving the overall quality of *pixian* douban.

### 3.2. Safety Indicators Analysis of Pixian Douban

**Figure 2:**
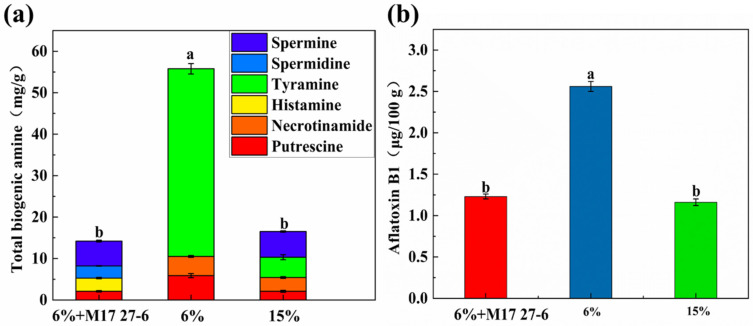
(**a**) Biogenic amine content of the “6% + M17 27-6”, “6%” and “15%” groups; (**b**) Aflatoxin B1 levels of the “6% + M17 27-6”, “6%” and “15%” groups. (a and b were represented significant differences between different groups.)

Biogenic amines are produced by bacterial decarboxylation of amino acids during the fermentation process. Excessive intake of biogenic amines not only causes nausea, vomiting, diarrhea, and abdominal pain but also rashes, itching, headaches, and high blood pressure [45]. Common biogenic amines in fermented foods include putrescine, histamine, tyramine, and others. A decrease in salinity during legume fermentation can increase biogenic amine content. As presented in Figure 2a, the biogenic amine content in *pixian* douban after 35 days of fermentation was significantly lower in the low-salt group inoculated with *P. polymyxa* M17 27-6 and in the high-salt control group compared to the uninoculated low-salt control group (*p* < 0.05). Putrescine was detected in all three groups, with the highest concentrations (5.88 ± 0.50 mg/100 g) found in the low-salt group. Histamine and spermidine were detected in the low-salt group inoculated with *P. polymyxa* M17 27-6; however, tyramine and necrotinamide were not detected. We inferred that in a low-salt environment, the introduction of *P. polymyxa* M17 27-6 may have inhibited the growth of harmful bacteria capable of producing tyramine and necrotinamide. However, the growth of certain harmful bacteria producing histamine and spermidine was only partially suppressed. Despite this, concentrations of both histamine and spermidine remained below 6 mg/100 g, meeting regulatory standards. In contrast, the highest concentrations of tyramine (45.28 mg/100 g) were detected in the uninoculated low-salt control group, which could cause disorders such as headache and hypertension when excessive [46]. Additionally, cadaverine, tyramine, and spermine were detected in the high-salt control group, all remaining below the threshold of 6 mg/100 g. These results show that biogenic amine accumulation during fermentation is linked to reduced salinity, with higher levels of cadaverine, histamine, and tyramine in low-salt products [47]. Thus, the results of this study are consistent with these observations. The biogenic amine concentration in the low-salt group inoculated with *P. polymyxa* M17 27-6 was significantly lower than in the uninoculated control, indicating that *P. polymyxa* M17 27-6 reduces biogenic amine production during fermentation.

Safety testing ensured the *pixian* douban met regulatory standards, with aflatoxin B1 content complying with the Chinese national standard (GB/T 20560-2006 [39]) limit of 5 μg/kg. To further evaluate microbial safety, foodborne pathogens such as *Salmonella*, *Staphylococcus*, *Escherichia,* and *Shigella* were tested in different *pixian* douban after fermentation, following Chinese national microbiological testing standards, as shown in Supplementary Table S3. Notably, no foodborne pathogens were detected in either the low-salt group inoculated with *P. polymyxa* M17 27-6 or the uninoculated high-salt control group. The antimicrobial compounds produced by *P. polymyxa* M17 27-6 effectively inhibited foodborne pathogenic bacteria and spoilage bacteria, thereby enhancing the safety profile of the low-salt *pixian* douban. In the high-salt group, the growth of foodborne pathogens and spoilage bacteria was suppressed due to the inhibitory effects of elevated salt concentrations.

### 3.3. Bioactive Compounds and Antioxidant Activity Analysis of Pixian Douban

**Figure 3:**
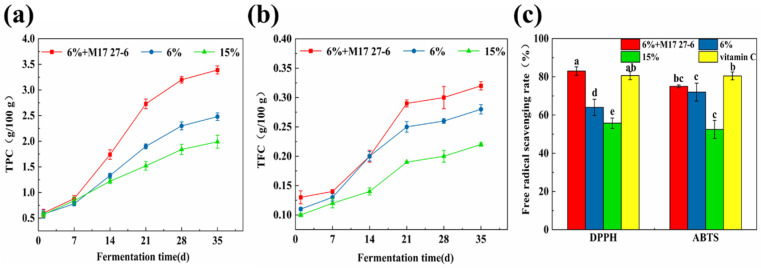
Changes in phenolics and flavonoids and antioxidant activity during fermentation of three groups of samples. (**a**) Total phenol content of the “6% + M17 27-6”, “6%” and “15%” groups; (**b**) Total flavonoid content of the “6% + M17 27-6”, “6%” and “15%” groups. (**c**) DPPH, ABTS activity of the “6% + M17 27-6”, “6%”, “15%” groups and vitamin C. (a, b, c, d and e were represented significant differences between different groups.)

Phenols and flavonoids, key bioactive components in *pixian* douban, promote human health. However, the impact of fermentation agents on their concentrations under low salinity is still unexplored. As shown in Figure 3a, the total phenol content in the low-salt group inoculated with *P. polymyxa* M17 27-6 increased from 0.60 ± 0.07 g/100 g initially to 3.39 ± 0.08 g/100 g after 35 d of fermentation, whereas the uninoculated high-salt group had the lowest content (1.99 ± 0.13 g/100 g). Phenolics form complexes with sugars and glycosides in plants through hydroxyl groups. Previous research showed similar increases in total phenol levels in fermented legumes using fermentation agents. Total flavonoids followed a comparable trend (Figure 3b), with the highest content in the inoculated low-salt group, followed by the uninoculated low-salt and high-salt groups. Consistent with findings by Kim et al. [33], low-salt (8%) *pixian* douban contained higher phenolics and flavonoids than high-salt versions. These results show that using a fermentation agent and reducing salinity mitigated the negative effects of excessive salt and enhanced bioactive components beneficial to human health.

In vitro antioxidant properties were further evaluated through DPPH and ABTS free radical scavenging activity. As shown in Figure 3c, significant differences (*p* < 0.05) were observed among the fermented *pixian* douban groups, with 0.1 mg/mL Vitamin C as the control. The DPPH scavenging activities for the “6% + M17 27-6”, “6%”, and “15%” groups were 82.99 ± 2.26%, 63.99 ± 4.21%, and 55.74 ± 2.71%, respectively, while ABTS scavenging activities were 74.99 ± 0.73%, 72.00 ± 4.68%, and 52.49 ± 4.73%. These findings underscore that the utilization of *P. polymyxa* M17 27-6 for fermentation under low salinity conditions enhances antioxidant activity. Based on our previous results measuring the DPPH and ABTS radical scavenging activity of the strains, we inferred that the higher activity observed in the low-salt inoculation group may be attributable to the inherent antioxidant activity of *P. polymyxa* M17 27-6 itself.

### 3.4. Organic Acids Analysis of Pixian Douban

**Figure 4:**
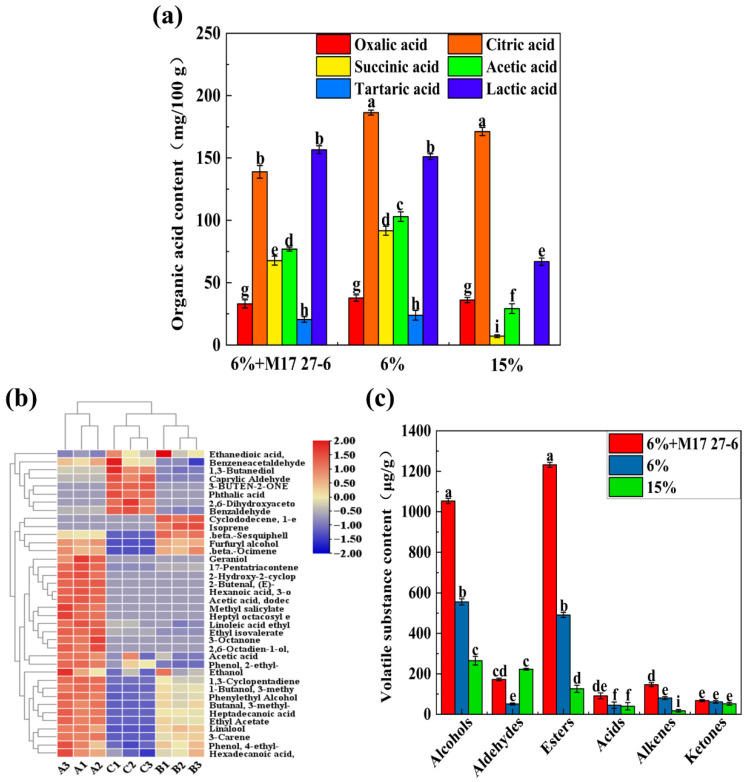
Volatile components in three groups of broad-bean paste by SPME-GC-MS. (**a**) Organic acid content of the “6% + M17 27-6”, “6%” and “15%” groups. (**b**) Heat map of volatile compound concentration and clustering. The color of the heat map transitions smoothly from a blue tint to a red tint. In this representation, the blue tint indicates lower concentrations of the compound, while the red tint signifies a gradual increase in the compound’s concentration. (A1, A2, A3 represent the low-salt inoculation group; B1, B2, B3 represent the low-salt control group; C1, C2, C3 represent the high-salt control group.) (**c**) Concentration of volatile compounds in each group. (a, b and c, etc. were represented significant differences between different groups.)

Organic acids generated during fermentation, hydrolysis, and microbial activity play a crucial role in shaping the flavor, chemical stability, pH, and overall quality of *pixian* douban, thereby necessitating their quantification [48]. As shown in Figure 4a, the organic acid content varied significantly among the three groups (*p* < 0.05). Specifically, oxalic, citric, succinic, acetic, tartaric, and lactic acids were detected across all groups, consistent with study of Lin et al. [49]. Among these acids, citric acid was the most abundant in *pixian* douban, followed by lactic acid. The citric acid content in the “6% + M17 27-6”, “6%”, and “15%” groups was 138.91 ± 5.07 mg/100 g, 186.40 ± 2.01 mg/100 g and 171.08 ± 3.24 mg/100 g, respectively, with the highest content in the uninoculated low-salt group. The lactic acid contents in the “6% + M17 27-6”, “6%”, and “15%” groups were 123.57 ± 6.57 mg/100 g, 146.80 ± 4.83 mg/100 g and 66.87 ± 2.93 mg/100 g, respectively. The citric acid to lactic acid ratio in the low-salt inoculated group was 1.12, compared to 1.22 in the low-salt non-inoculated group. In contrast, the high-salt group exhibited a ratio of 2.06. These results suggest that the low-salt inoculated group produced relatively more lactic acid, likely due to the metabolic characteristics of *P. polymyxa* M17 27-6, which likely promoted lactic acid synthesis by lactic acid bacteria and influenced the overall acid profile. Furthermore, the high-salt group showed significantly lower lactic acid content than the low-salt group, suggesting that the high-salt environment inhibited lactic acid bacteria activity, leading to reduced lactic acid synthesis. Changes in organic acids directly affected the sensory characteristics of *pixian* douban. The higher lactic acid content in the low-salt inoculation group may correlate with its milder acidity and more balanced flavor profile, which was consistent with sensory evaluations that described it as “softer in texture” and “less acidic.” In contrast, the high-salt group exhibited significantly lower lactic acid levels, contributing to its sensory characteristics of “stronger saltiness” and “more somewhat astringent.”

### 3.5. Volatile Compounds Analysis of Pixian Douban

#### 3.5.1. Volatile Compounds Analyzed by SPME-GC-MS

SPME-GC-MS analysis identified a total of 38 volatile compounds in *pixian* douban samples fermented for 35 d, including 8 alcohols, 5 aldehydes, 11 esters, 3 acids, 6 alkenes, 3 ketones, and 2 phenols (Supplementary Table S4). The low-salt group inoculated with *P. polymyxa* M17 27-6 had 32 volatile compounds, compared to 24 in the low-salt control and 17 in the high-salt control. The low-salt samples showed higher levels and diversity of volatile compounds, likely due to increased microbial activity. The finding that low-salt doubanjiang exhibits richer flavor is consistent with the results previously reported by Hee et al. in their study on the effects of varying salt concentrations on Korean doenjang [2]. As illustrated in Figure 4b, esters and alcohols were predominant in all groups, while ketones and alkenes were less abundant, consistent with Yang et al. [50]. The high-salt group, in particular, exhibited the lowest volatile content, as elevated salinity inhibits functional bacterial growth, leading to inefficient fermentation. Typically, extended fermentation periods ranging from 6 months to 2 years are required for robust flavor development [51,52], making it challenging to generate a wide array of flavor compounds within just 35 d of fermentation.

In this study, heat variation analysis of volatile compounds (Figure 4c) revealed significant differences among the three *pixian* douban groups. Esters were the most abundant volatiles, likely formed through microbial enzyme-catalyzed reactions or non-enzymatic esterification during fermentation. Key esters, such as ethyl palmitate, ethyl linoleate, ethyl acetate, isoamyl acetate, and butyl acetate, contributed to fruity aromas. Among these, the primary fruit aromas in the low-salt group originated from ethyl acetate (OAV = 5.3) and ethyl isovalerate (OAV = 3.2). The OAV values of these compounds exceeded 1, indicating their dominant role in the aroma profile of the low-salt group. Alcohols, the second most detected group, were primarily produced via yeast metabolism of sugars or reduction in aldehydes and ketones under low redox potential. Among the seven alcohols isolated, phenylethanol had the highest content, with a rose-honey-like odor and imparting a grassy, woody, vegetal, greenish flavor to the *pixian* douban. In the low-salt group inoculated with *P. polymyxa* M17 27-6, Linalool (OVA = 4.1), an aromatic compound with a pleasant fragrance, was also detected. It imparts a more pronounced herbal aroma to the low-salt fermentation. Aldehydes, including 3-methylbutyraldehyde, benzaldehyde, and phenylacetaldehyde, were also identified. The content of benzaldehyde was higher in *pixian* douban, with a soy sauce-like aroma, which was produced by lactobacillus metabolism [50], while phenylacetaldehyde, with a sweet flavor, resulted from lipid oxidation [53]. Overall, low-salt *pixian* douban exhibited higher flavor compound levels than the high-salt group, and inoculation with *P. polymyxa* M17 27-6 enhanced *pixian* douban quality and flavor production.

#### 3.5.2. Volatile Compounds Analyzed by HS-GC-IMS

**Figure 5:**
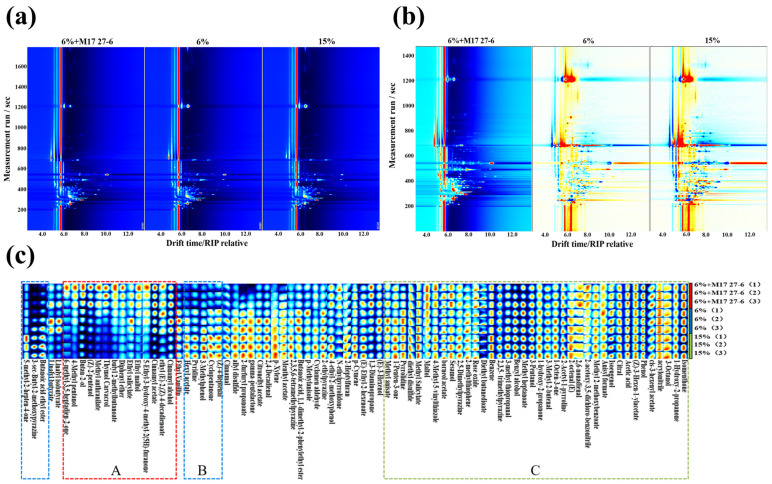
HS-GC-IMS analysis of volatile flavor substances in low-salt and high-salt broad-bean paste. (**a**) 2D topography. The red vertical line at 6.0 on the horizontal axis signifies the normalized reactive ion peak (RIP), with each point on either side representing a volatile compound. The concentration of volatile compounds was conveyed in color, with more intense hues signifying higher concentration. White symbolizes low concentration, while red signifies high concentration. (**b**) 2D difference comparison. (**c**) Fingerprints of volatile compounds. In each sample, represented by three parallels, each dot in a row signifies a detected compound, while each dot in a column represents the signal peak of the same compound in different broad-bean pastes. A brighter color indicates a greater peak intensity and higher concentration, and vice versa.

A total of 83 volatile compounds were identified using GC-IMS and the NIST/IMS databases, including 24 esters, 11 alcohols, 11 aromatics, 10 aldehydes, 8 ketones, 6 pyrazines, 4 ethers, 1 acid, 1 terpene, 1 furan, and 6 others. Among these compounds, esters and alcohols were the most abundant, serving as the primary aroma compounds in fermented *pixian* douban. The migration times and retention indices of each compound were analyzed for qualitative comparison (Supplementary Table S5). For visual comparison, the low-salt group inoculated with *P. polymyxa* M17 27-6 was used as the reference sample. The spectra of the *pixian* douban were characterized by subtracting the signals of the reference samples; the final comparison results are shown in Figure 5a,b.

To further analyze differences in compound types and concentrations among the three *pixian* douban groups, the Gallery Plot plugin was used to generate fingerprints (Figure 5c). Zone A (red box) highlighted volatile compounds with higher concentrations in the low-salt group inoculated with *P. polymyxa* M17 27-6, including Cinnamyl Alcohol, (Z)-2-pentenol, and Butan-2-ol. Cinnamyl Alcohol and Cinnamyl Acetate impart fruity, floral, and sweet flavors, balancing the *pixian* douban’s salty and spicy notes [54]. Ethyl maltol, Butyl 2-methylbutyrate and 6-methyl-3,5-pentadien-2 contribute apple, herbal, and creamy flavors, while Thymol, Carvacrol, and (Z)-2-pentenol add herbal, spicy, and mellow notes [55]. Zone B (blue box) identified compounds in the high-salt group without *P. polymyxa* M17 27-6, such as (Z)-4-heptenal, 3-Methylphenol, and 3-sec-Butyl-2-methoxypyrazine. These compounds contribute herbaceous, smoky, burnt, nutty, and baking aromas to the *pixian* douban, with 5-methyl-2-hepten-4-one adding pungent, fruity, and herbaceous flavors [56,57,58,59]. Although the low-salt and high-salt groups contained distinct volatile compounds, their overall flavor profiles remained similar due to overlapping aromatic characteristics.

Area C (green box) identified volatile compounds common to all three groups. Acetic acid, the only detected acid, adds a sour flavor and is produced by Lactobacillus or carbohydrate metabolism during fermentation [57]. Alcohols and esters were the most abundant compounds across all groups. Alcohols, primarily derived from sugar and amino acid metabolism, included 3-Octanol, which imparts a mushroom-like flavor [60], and benzyl alcohol, which contributes floral and honey-like aromas [61]. Esters, formed from saturated fatty acids, add floral, fruity, and sweet notes [62], while methyl esters and ethyl esters accounted for the largest proportion, such as 2-furfuryl propanoate, Ethyl-2,4-decadienoate, Ethyl-2-hexenoate, and diethyl butanedioate. Aldehydes, the third most abundant group, included cyclamen aldehyde, which offers a fruity sweetness and floral fragrance reminiscent of lilacs and lilies. Overall, GC-MS and GC-IMS results indicated that the low-salt group releases a greater diversity of volatile compounds.

### 3.6. Microbial Diversity Analysis of Pixian Douban

**Figure 6:**
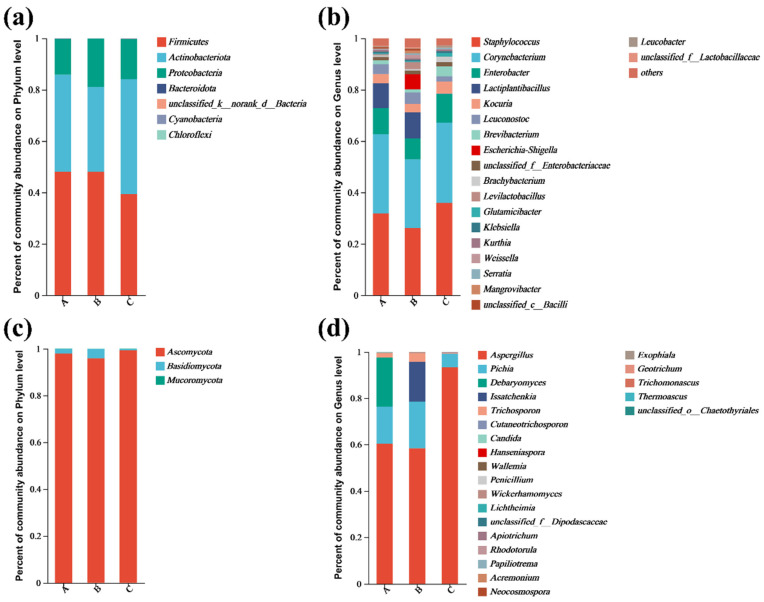
The composition of bacterial and fungal communities at the end of fermentation of broad-bean paste. A: 6% + M17 27-6; B: 6%; C: 15%. (**a**) Relative abundance at the bacterial phylum level. (**b**) Relative abundance at the bacterial genus level. (**c**) Relative abundance at the fungal phylum level. (**d**) Relative abundance at the fungal genus level.

The bacterial and fungal community composition at the end of *pixian* douban fermentation was analyzed at different taxonomic levels, with results shown in Figure 6a–d.

At the phylum level (Figure 6a), *Firmicutes*, *Actinobacteria*, and *Proteobacteria* dominated the bacterial communities in all three *pixian* douban groups, consistent with previous studies [63]. At the genus level (Figure 6b), *Bacillus*, *Staphylococcus*, and *Corynebacterium* were dominant across all salinity levels, with Staphylococcus and Corynebacterium contributing significantly to the fermentation process [64]. Lactobacillus was abundant in low-salt samples but absent in high-salt ones due to salt inhibition, which corresponded with lower pH and higher acid content in low-salt groups [65]. The uninoculated low-salt group exhibited higher levels of *Escherichia* and *Shigella*, pathogens associated with foodborne illnesses. Inoculation with *P. polymyxa* M17 27-6, however, eliminated these pathogens and reduced spoilage risks, indicating its antimicrobial efficacy. Significant differences (*p* < 0.05) in species composition among the groups are shown in Supplementary Figure S2. In addition to the high content of *Staphylococcus*, *Bacillus*, and *Lactobacillus* in the *pixian* douban of the low-salt group, the uninoculated low-salt control group also contained high levels of *Escherichia coli*, *Listeria monocytogenes*, and *Shigella Castellani*, posing safety risks. Inoculation with *P. polymyxa* M17 27-6, however, eliminated these pathogens and reduced spoilage risks, indicating its antimicrobial efficacy.

Additionally, fungal diversity was lower compared to bacteria, with Ascomycota and Basidiomycota being the dominant phyla [4]. The microbial composition of the three groups of the fungal genus is shown in Figure 6d, with the presence of *Aspergillus*, *Pichia*, *Debaryomyces*, and *Trichosporon* in all three samples. Compared to the low-salt control group, the abundance of *Debaryomyces* was high in the low-salt group inoculated with *P. polymyxa* M17 27-6. The inoculation with *P. polymyxa* M17 27-6 enhanced the abundance of *Debaryomyces*, which contributed to organic acid and ester production, boosting flavor [66]. The result was also aligned with the previously mentioned finding that the low-salt inoculation group exhibited higher levels of flavor compounds. However, *Issatschenkia* and *Trichosporons* in the low-salt uninoculated group exhibited higher abundance than low-salt group inoculated with *P. polymyxa* M17 27-6. *Issatchenkia* was generally not considered a beneficial bacterium in the fermentation process of *pixian* douban. Excessive *Issatchenkia* can produce large amounts of acid, thereby destroying the rich flavor of *pixian* douban [18]. Therefore, this finding was consistent with the earlier results indicating higher total acid and organic acid content in the uninoculated low-salt group. The presence of harmful fungi like Issatschenkia was significantly reduced in the inoculated low-salt group. In contrast, high salt levels inhibited both bacterial and fungal growth, reducing the overall microbial diversity.

### 3.7. Electronic Sensory Analysis of Pixian Douban

**Figure 7:**
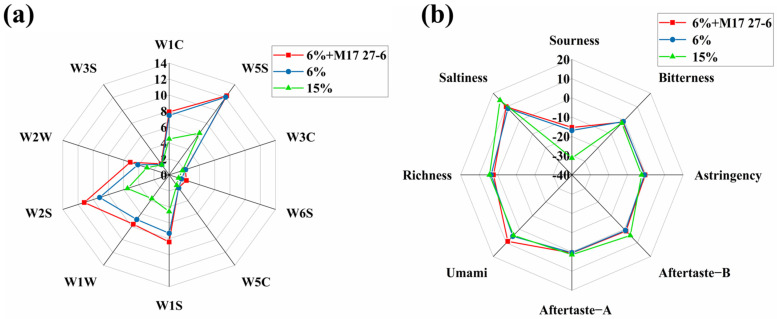
Radar plots of three groups of broad-bean paste analyzed by electronic analysis. (**a**) Radar plot for the electronic nose analysis. (**b**) Radar plot for the electronic tongue analysis.

The results of electronic nose are displayed in Figure 7a. While the radar charts of the three groups displayed similar sensor signal patterns, differences in response values were observed. The W2S sensor, sensitive to organic solvents, detected common aroma compounds (alcohols, aldehydes, ketones, esters, etc.) in *pixian* douban, aligning with GC-MS results. The radar chart revealed that the low-salt group inoculated with *P. polymyxa* M17 27-6 exhibited a more intense aroma, indicating that *P. polymyxa* M17 27-6 effectively enhances *pixian* douban flavor.

The results of the electronic tongue detection of the *pixian* douban are shown in Figure 7b. Significant changes (*p* < 0.05) occurred in the oral sensation of the low-salt group inoculated with *P. polymyxa* M17 27-6. The uninoculated low-salt control group exhibited a stronger acidic flavor, consistent with its higher total acid content. In contrast, the low-salt group inoculated with *P. polymyxa* M17 27-6 showed enhanced freshness, whereas the uninoculated high-salt group had a more pronounced salty taste. Freshness, a key flavor attribute in *pixian* douban, was more appealing to consumers. Thus, *pixian* douban inoculated with *P. polymyxa* M17 27-6 had a better flavor.

## 4. Conclusions

In this study, *Paenibacillus polymyxa* M17 27-6, selected for its antimicrobial production, safety, and fermentation efficiency, was used to ferment low-salt *pixian* douban. Compared to uninoculated low-salt *pixian* douban, inoculation with *P. polymyxa* M17 27-6 significantly reduced aflatoxin B1 and biogenic amines, enhancing the safety of the product. In addition, the inoculated group showed reduced risk of spoilage and pathogenicity. Specifically, *P. polymyxa* M17 27-6 inhibited the growth of pathogenic *Escherichia* and *Shigella* while also suppressing *Issatchenkia* and *Trichosporon* species, which could cause spoilage. Furthermore, the inoculation led to increased levels of amino acid nitrogen, total acids, organic acids, and volatile compounds compared to the high-salt control. Overall, this study supports the use of *P. polymyxa* M17 27-6 to improve the safety, quality, and fermentation efficiency of low-salt *pixian* douban. This approach could be applied in industrial-scale production, though further investigation of fermentation stability and control of quality and safety is needed. Future studies should focus on potential sources of variation, such as biological variability, raw material differences, and environmental factors, to better understand the interactions between *P. polymyxa* M17 27-6 and other microbial communities during fermentation.

## Data Availability

The original contributions presented in the study are included in the article/Supplementary Materials. Further inquiries can be directed to the corresponding authors.

## Supplementary Materials

The following supporting information can be downloaded at https://www.mdpi.com/article/10.3390/foods14244200/s1. Figure S1. Antimicrobial zone diagram of *Paenibacillus polymyxa* M17 27-6 against various pathogenic bacteria. Figure S2. The differences in species composition among the three groups of samples. A: 6% ± M17 27-6; B: 6%; C: 15%. Figure S3. Extracellular enzyme hydrolysis circle diagram of *P. polymyxa* M17 27-6. Table S1. Gradient elution procedure for the determination of biogenic amines by HPLC. Table S2. The performance of ten metal–oxide–semiconductor sensors in electronic nose. Table S3. Detection of pathogenic bacteria after fermentation of Bean paste. Table S4. Volatile components in three groups of broad-bean paste by SPME-GC-MS. Table S5. The migration times and retention indices of each compound in three groups in bean paste by HS-GC-IMS. Table S6. Results of drug sensitivity tests and antibiotic susceptibility of *Paenibacillus polymyxa* M17 27-6. Table S7. Results of extracellular enzyme activity assay of *Paenibacillus polymyxa* M17 27-6.
