# Supplementary material for: Enhancement of Quality and Safety of Low-Salt Pixian Douban Fermentation with Paenibacillus polymyxa M17 27-6
Source: Foods. 2025 Dec 7;14(24):4200. doi: 10.3390/foods14244200 (PMC12732237; doi:10.3390/foods14244200)
Supplement: Supplementary file 1 [file foods-14-04200-s001.zip › Supplementary materials.pdf]

Improving Quality and Safety of Pixian Douban Through Low-Salt Fermentation with *Paenibacillus polymyxa* M17 27-6

Zirong Gao[a], Weihong Tao[a], Xiaolei Ren[a], Ningbo Qin[a], Yingxi Chen[a], Chaofan Ji[a], Xinping Lin[a], Yiwei Dai[a, *], Sufang Zhang[a, *]

[a]SKL of Marine Food Processing & Safety Control, National Engineering Research Center of Seafood, Collaborative Innovation Center of Seafood Deep Processing, School of Food Science and Technology, Dalian Polytechnic University, Dalian, Liaoning, 116034, China

*Corresponding author: Sufang Zhang & Yiwei Dai

E-mail:

Sufang Zhang: zhangsf@dlpu.edu.cn

Yiwei Dai: ywdai6228@126.com

Author email:

ZiRong Gao: zrgao1015@163.com

Weihong Tao: twh12345600@163.com

Xiaolei Ren: 1255816058@qq.com

Ningbo Qin: qinningbo6902765@163.com

Yingxi Chen: yingxichen24@163.com

Chaofan Ji: Jichaofan@outlook.com

Xinping Lin: yingchaer@163.com

Supplementary Table S1 Gradient elution procedure for the determination of biogenic amines by HPLC

Elution time /min	Mobile phase A (%)	Mobile phase B (%)
0	45	55
10	35	65
15	20	80
20	20	80
25	10	90
30	10	90
35	45	55

Supplementary Table S2 The performance of ten metal-oxide-semiconductor sensors in electronic nose

Sensor name	Sensor sensitives
W1C	Aromatic organic compounds
W5S	Nitrogen oxides
W3C	Ammonia, aromatic compounds
W6S	Hydrogens
W5C	Short-chain alkanes, aromatic compounds
W1S	Methyl compounds
W1W	Inorganic sulfides
W2S	Alcohols, aldehydes, ketones
W2W	Organic sulfides
W3S	Long-chain alkanes

Supplementary Table S3 Detection of pathogenic bacteria after fermentation of Bean paste

Pathogenic bacteria	log CFU/g		
	6%+M17 27-6	6%	15%
Escherichia coli	ND	4.2±1.2^{a}	ND
Salmonella	ND	ND	ND
Shigella Castellani	ND	2.7±0.9^{b}	ND
Staphylococcus aureus	ND	2.0±0.5^{c}	ND

Note: *Different superscript letters indicate statistical differences ($p<0.05$);

All values are means ± standard deviation from triplicate analyses.

Supplementary Table S4 volatile components in three groups of broad-bean paste by SPME-GC-MS

Compounds	molecular formula	Concentration (μg/g)			Odor description
		6%+M17 27-6	6%	15%	
**Esters**					
Ethyl Acetate	$C_4H_8O_2$	629.21±40.14[a]	322.83±54.48[b]	4.26±0.82[c]	Brandy, fruity
1-Butanol, 3-methyl-, acetate	$C_7H_{14}O_2$	68.20±2.23[a]	32.72±4.53[b]	ND	Pear
Methyl salicylate	$C_8H_8O_3$	22.33±3.25[a]	ND	ND	
Ethanedioic acid, bis(trimethylsilyl) ester	$C_8H_{18}O_4Si_2$	15.51±0.49[a]	21.53±5.49[a]	19.72±2.76[a]	
Ethyl isovalerate	$C_7H_{14}O_2$	69.57±3.43[a]	ND	8.13±0.92[b]	Apple, vinous
Hexanoic acid, 3-oxo-, methyl ester	$C_7H_{12}O_3$	50.13±2.17[a]	ND	ND	Pineapple
Acetic acid, dodecyl ester	$C_{14}H_{28}O_2$	51.77±1.97[a]	ND	ND	
Heptyl octacosyl ether	$C_{35}H_{72}O$	33.80±4.30[a]	ND	ND	Flower
17-Pentatriacontene	$C_{35}H_{70}$	89.73±6.82[a]	13.27±1.38[b]	ND	
Hexadecanoic acid, ethyl ester	$C_{18}H_{36}O_2$	67.48±7.46[a]	53.15±6.13[b]	34.53±6.75[c]	Cherries, blackcurrant
Linoleic acid ethyl ester	$C_{20}H_{36}O_2$	134.25±5.80[a]	47.48±6.59[b]	59.47±6.57[b]	
**Alcohols**					
Ethanol	C_2H_6O	61.93±14.47[a]	49.56±15.45[ab]	34.69±6.81[b]	
Furfuryl alcohol	$C_5H_6O_2$	33.06±1.87[a]	30.23±1.87[a]	ND	
Linalool	$C_{10}H_{18}O$	63.00±0.51[a]	45.74±3.67[b]	ND	Flower
Phenylethyl alcohol	$C_8H_{10}O$	717.56±37.79[a]	412.42±57.78[b]	146.52±24.68[c]	Apple, pear, cocoa, honey
3-Octanone	$C_8H_{16}O$	18.32±2.70[a]	ND	ND	Fruity
Geraniol	$C_{10}H_{18}O$	53.83±12.45[a]	ND	ND	Flower
2,6-Octadien-1-ol, 3,7-dimethyl-	$C_{10}H_{18}O$	68.84±8.55[a]	ND	ND	
1,3-Butanediol	$C_4H_{10}O_2$	36.75±0.93[b]	17.45±2.87[c]	83.74±15.92[a]	

**Aldehydes**					
Butanal, 3-methyl-	$C_5H_{10}O$	9.26±0.68[a]	5.10±0.96[b]	ND	
2-Butenal, (E)-	C_4H_6O	61.33±1.88[a]	ND	ND	
Benzaldehyde	C_7H_6O	37.44±1.31[b]	16.79±3.49[c]	129.09±10.75[a]	Almond
Benzeneacetaldehyde	C_8H_8O	57.36±4.99[ab]	29.02±7.48[b]	62.36±22.48[a]	Hyacinth
Caprylic aldehyde	$C_8H_{16}O$	6.75±0.33[b]	ND	31.44±4.41[a]	Fruity
**Alkenes**					
1,3-Cyclopentadiene, 5-ethenyl-5-methyl-	C_5H_6	24.66±0.43[a]	9.22±1.82[b]	ND	
2-Hydroxy-2-cyclopenten-1-one	$C_5H_6O_2$	5.63±0.51[a]	ND	ND	Maple
Beta-Ocimene	$C_{10}H_{16}$	67.73±7.20[a]	68.11±8.75[a]	ND	Herbal
3-Carene	$C_{10}H_{16}$	20.99±0.56[a]	12.36±2.05[b]	ND	Pine wood
Beta-Sesquiphellandrene	$C_{15}H_{24}$	18.44±1.52[b]	33.87±2.71[a]	ND	Lemon
Isoprene	C_5H_8	ND	55.04±7.90[a]	ND	
**Acids**					
Acetic acid	$C_2H_4O_2$	31.63±1.45[a]	15.07±3.12[b]	19.17±1.16[ab]	
Heptadecanoic acid	$C_{17}H_{34}O_2$	59.52±5.14[a]	29.93±2.11[b]	ND	
Phthalic acid	$C_8H_6O_4$	ND	ND	20.66±1.98[a]	
**ketones**					
Cyclododecene, 1-ethynyl-2-methyl-, (E)-	$C_{15}H_{24}$	ND	11.65±0.92[a]	ND	
3-Buten-2-one	$C_{13}H_{20}O$	ND	ND	17.27±0.41[a]	Spicy
2,6-Dihydroxyacetophenone	$C_8H_8O_3$	ND	ND	30.37±5.60[a]	
**Phenols**					
Phenol,4-ethyl-	$C_8H_{10}O$	129.22±24.23[a]	99.64±10.83[a]	36.27±6.92[b]	Sweet
Phenol,2-ethyl-	$C_8H_{10}O$	137.94±6.51[a]	14.44±4.46[c]	64.13±2.20[b]	Sweet

Data was expressed as average value ± standard deviation; ND indicates not detected;

Values marked with different letters in the same row indicated significant differences ($p < 0.05$, $n = 3$).

Supplementary Table S5 The migration times and retention indices of each compound in three groups in bean paste by HS-GC-IMS

Compounds	CAS#	Formula	MW	RI	Rt (s)	Dt (ms)	Odor description	Comment
**Alcohols**								
Benzyl alcohol	100-51-6	C7H8O	108.1	1883.4	1214.097	1.15835	sweet, flower	
3-Octanol	589-98-0	C8H18O	130.2	1414.2	493.189	1.39787		
(E)-3-Hexenol	928-97-2	C6H12O	100.2	1364.5	437.652	1.2435	moss, fresh	
(Z)-2-Pentenol	1576-95-0	C5H10O	86.1	1326.2	395.321	1.61794		
Cinnamyl alcohol	104-54-1	C9H10O	134.2	1274.4	350.161	1.56581	oil	
4-Methyl pentanol	626-89-1	C6H14O	102.2	1274.6	350.289	1.31678		
1-Hydroxy-2-propanone	116-09-6	C3H6O2	74.1	1284.4	356.469	1.042	sweet, fruit	Monomer
1-Hydroxy-2-propanone(D)	116-09-6	C3H6O2	74.1	1342.6	412.906	1.04294	sweet, fruit	Dimer
3-Pentanol	584-02-1	C5H12O	88.1	1121.8	269.87	1.19798		
Butan-2-ol	78-92-2	C4H10O	74.1	1077.2	251.169	1.15148	wine, sweet	
Maltol	118-71-8	C6H6O3	126.1	1109.8	264.706	1.15105	caramel	
**Esters**								
Cinnamyl acetate	103-54-8	C11H12O2	176.2	1465.4	544.148	1.75058		
Menthyl acetate	89-48-5	C12H22O2	198.3	1587.3	687.705	1.23829		
gamma-Pentalactone	108-29-2	C5H8O2	100.1	1584.1	683.371	1.41666	milk	
2-Furfuryl propanoate	623-19-8	C8H10O3	154.2	1587.7	688.211	1.48555	caramel, fruit	
Butanoic acid, 1,1-dimethyl-2-phenylethyl	10094-34-5	C14H20O2	220.3	1477.8	557.219	1.18268	Flower, fruit	

ester							
Ethyl (E)-2, (Z)-4-decadienoate	3025-30-7	C12H20O2	196.3	1472.8	551.926	1.54727	
Methyl anisate	121-98-2	C9H10O3	166.2	1379	454.937	1.26401	
Linalyl butyrate	78-36-4	C14H24O2	224.3	1379.2	455.144	1.65677	pear, sweet
Linalyl isobutyrate	78-35-3	C14H24O2	224.3	1364	437.148	1.65185	
cis-3-hexenyl acetate	3681-71-8	C8H14O2	142.2	1355.3	427.106	1.04646	
(E)-Ethyl-2-hexenoate	27829-72-7	C8H14O2	142.2	1335.3	405.022	1.3223	
Anisyl formate	122-91-8	C9H10O3	166.2	1341.3	411.487	1.08172	
Methyl 2-methoxybenzoate	606-45-1	C9H10O3	166.2	1337.3	407.1	1.2223	
Methyl heptanoate	106-73-0	C8H16O2	144.2	1296.2	365.01	1.36299	Apple, pear
Methyl anthranilate	134-20-3	C8H9NO2	151.2	1296.4	365.139	1.26029	honey, flower
Citronellyl acetate	150-84-5	C12H22O2	198.3	1313.2	381.908	1.47029	rose, dust
Butyl 2-methylbutanoate	15706-73-7	C9H18O2	158.2	1274.4	350.161	1.37205	
Ethyl salicylate	118-61-6	C9H10O3	166.2	1263.7	343.505	1.26152	wintergreen, mint
Borneol acetate	76-49-3	C12H20O2	196.3	1293.8	362.706	1.22239	
(Z)-3-Hexen-1-ylacetate	3681-71-8	C8H14O2	142.2	1310.7	379.339	1.04276	
Methyl Salicylate	119-36-8	C8H8O3	152.1	1230	323.363	1.15948	Peppermint
Diethyl butanedioate	123-25-1	C8H14O4	174.2	1193.5	302.896	1.2965	
Butanoic acid ethyl ester	105-54-4	C6H12O2	116.2	1092.2	257.31	1.20007	
Hexyl acetate	142-92-7	C8H16O2	144.2	1239.3	328.828	1.37919	Fruit, herb
**Ketones**							
6-methyl-3,5-heptadien-2-one	1604-28-0	C8H12O	124.2	1582.7	681.579	1.19908	

1-Octen-3-one	4312-99-6	C8H14O	126.2	1313.1	381.78	1.27625	
Isomenthone	491-07-6	C10H18O	154.3	1178.3	295.525	1.33984	
Cyclopentanone	120-92-3	C5H8O	84.1	1189.5	300.915	1.11582	
5-methyl-2-hepten-4-one	81925-81-7	C8H14O	126.2	981.4	215.303	1.23151	
1-Penten-3-one	1629-58-9	C5H8O	84.1	1050	240.423	1.09069	
N-ethylpyrrolidone	2687-91-4	C6H11NO	113.2	1141.1	278.383	1.15191	
5-Ethyl-3-hydroxy-4-methyl-2(5H)-furanone	698-10-2	C7H10O3	142.2	1239.2	328.726	1.27023	
**Acids**							
Acetic acid	64-19-7	C2H4O2	60.1	1442.1	520.338	1.04577	sour
**Furans**							
2-Heptylfuran	3777-71-7	C11H18O	166.3	1213.4	313.897	1.40888	
**Aldehydes**							
3-Methyl thiopropanal	3268-49-3	C4H8OS	104.2	1428.4	506.843	1.09399	
Cyclamen aldehyde	103-95-7	C13H18O	190.3	1427.9	506.341	1.35326	
Citral	5392-40-5	C10H16O	152.2	1394.6	474.178	1.04672	lemon
2,4-Decadienal	2363-88-4	C10H16O	152.2	1333.4	402.944	1.42325	seaweed
Cinnamal	104-55-2	C9H8O	132.2	1237.9	328.001	1.23755	
2-Octenal (E)	2548-87-0	C8H14O	126.2	1214	314.227	1.32808	
2,6-Nonadienal	557-48-2	C9H14O	138.2	1214	314.227	1.37656	cucumber, wax, green
(Z)-4-Heptenal	6728-31-0	C7H12O	112.2	1188.4	300.365	1.14446	
3-Methyl-2-butenal	107-86-8	C5H8O	84.1	1166.1	289.804	1.09745	
Ethyl Vanillin	121-32-4	C9H10O3	166.2	1417.3	496.088	1.73252	
**Terpenoids**							
Thymol Carvacrol	89-83-8	C10H14O	150.2	1587.5	687.939	1.27471	

**Ethers**							
Diphenyl ether	101-84-8	C12H10O	170.2	1416	494.933	1.29208	
Rose oxide	16409-43-1	C10H18O	154.3	1414.2	493.189	1.36286	flower
Diethyl disulfide	110-81-6	C4H10S2	122.2	1253.5	337.277	1.13977	
p-Methylanisole	104-93-8	C8H10O	122.2	1474.9	554.159	1.10954	
**Aromatics**							
Phenol	108-95-2	C6H6O	94.1	1883.7	1214.96	1.05443	phenol
Isoeugenol	97-54-1	C10H12O2	164.2	1466.8	545.606	1.30742	flower
Sesamol	533-31-3	C7H6O3	138.1	1350	421.093	1.19171	
4-Ethyl-2-methoxyphenol	2785-89-9	C9H12O2	152.2	1350.7	421.857	1.25114	
p-Cymene	99-87-6	C10H14	134.2	1325.6	394.632	1.18267	solvent, gasoline, citrus
Pyridine	110-86-1	C5H5N	79.1	1189.9	301.107	1.03225	
Ethyl maltol	4940-11-8	C7H8O3	140.1	1199.1	305.989	1.19758	
Benzene	71-43-2	C6H6	78.1	981.4	215.303	1.06722	
3-Methylphenol	108-39-4	C7H8O	108.1	1102.9	261.775	1.11584	
p-Xylene	106-42-3	C8H10	106.2	1154.1	284.255	1.04379	
2-Methylthiophene	554-14-3	C5H6S	98.2	1129.8	273.337	1.03369	
**Pyrazines**							
2,3,5,6-Tetramethylpyrazine	1124-11-4	C8H12N2	136.2	1457.2	535.626	1.20786	
2,3,5- Trimethylpyrazine	14667-55-1	C7H10N2	122.2	1421.3	499.973	1.1697	
2,5-Dimethylpyrazine	123-32-0	C6H8N2	108.1	1354.1	425.779	1.11432	
2-Ethylpyrazine	13925-00-3	C6H8N2	108.1	1321.1	389.958	1.15563	
2-Acetoxy-3,5-dichloro-benzonitrile	54300-08-2	C9H5Cl2NO2	230	1217.5	316.212	1.26848	

3-sec-Butyl-2-methoxypyrazine	24168-70-5	C9H14N2O	166.2	1090.9	256.751	1.28473	
**Others**							
1,3-Diaminopropane	109-76-2	C3H10N2	74.1	1326.2	395.324	1.29589	
2-Acetyl-1-pyrroline	85213-22-5	C6H9NO	111.1	1305.2	373.843	1.12889	
4-Methyl-5-vinylthiazole	1759-28-0	C6H7NS	125.2	1017.8	228.282	1.13679	
Pyrrolidine	123-75-1	C4H9N	71.1	1056.1	242.796	1.04123	alkaline
Allyl disulfide	2179-57-9	C6H10S2	146.3	1437.7	515.922	1.19712	
Acrylonitrile	107-13-1	C3H3N	53.1	1019.1	228.762	1.0932	

Note: MW represents the molecular weight of the volatile; RI, Rt and Dt represent the retention index, retention time and drift time of the volatile compound in GC-IMS, respectively;

Supplementary Table S6 Results of drug sensitivity tests and antibiotic susceptibility of *Paenibacillus polymyxa* M17 27-6.

Antibiotics	*Paenibacillus polymyxa* M17 27-6	
	Diameter of the Inhibition Zone (mm)	Sensitivity*
Cefazolin	36.60 ± 1.92	S
Erythromycin	36.83 ± 1.00	S
Gentamicin	36.20 ± 0.87	S
Tetracycline	47.33 ± 1.21	S
Amikacin	33.83 ± 0.49	S
Ampicillin	35.60 ± 0.35	S
Streptomycin	26.03 ± 1.20	S
Vancomycin	32.97 ± 0.40	S
Minocycline	45.83 ± 1.39	S
Cefalexin	23.70 ± 1.01	S
Penicillin G	42.33 ± 1.14	S

Supplementary Table S7 Results of extracellular enzyme activity assay of *Paenibacillus polymyxa* M17 27-6.

	Enzyme activity (U/mL)
Protease	192.12 ± 0.12
Amylase	123.42 ± 0.34
Cellulase	40.34 ± 0.12
Pectinase	65.72 ± 0.20

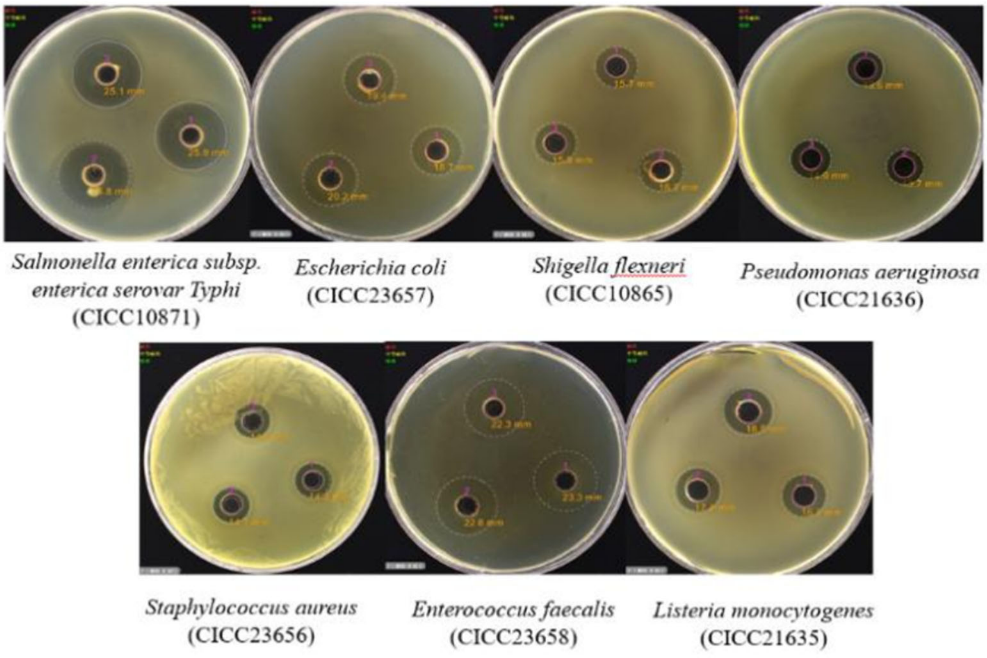

Supplementary Figure S1. Antimicrobial zone diagram of *Paenibacillus polymyxa* M17 27-6 against various pathogenic bacteria

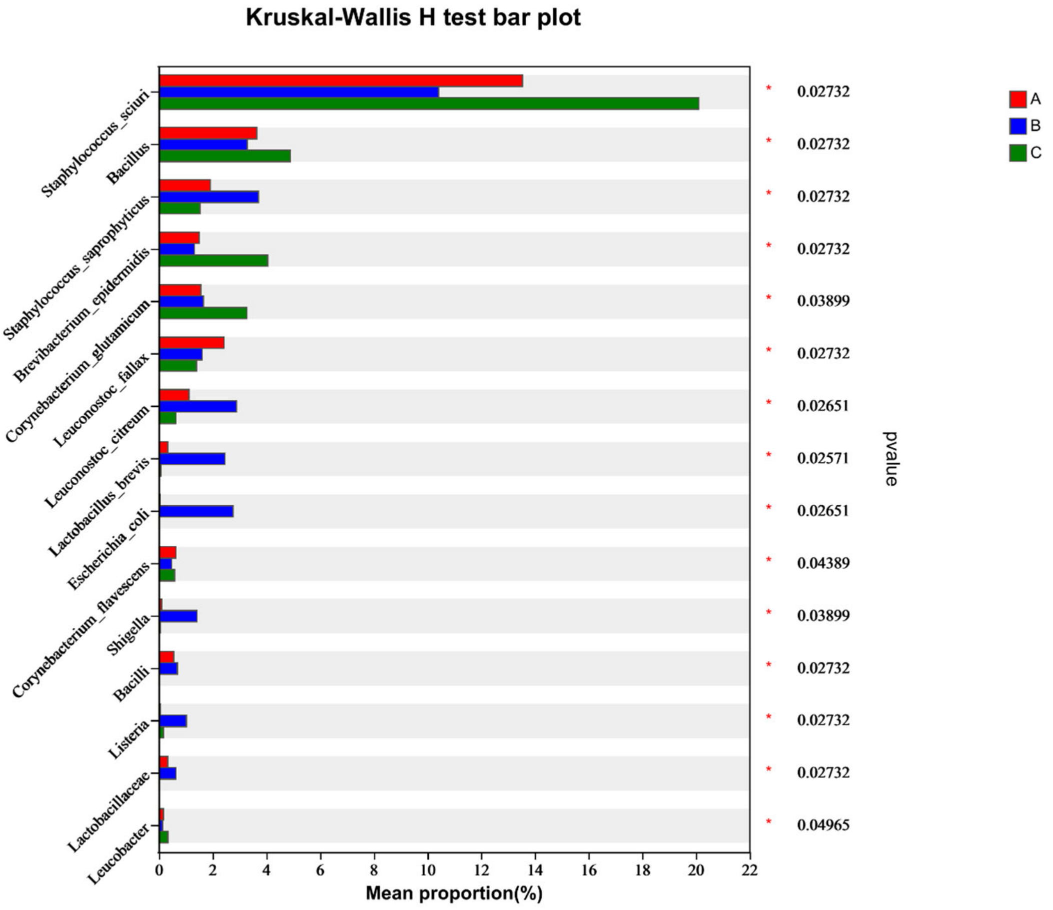

Supplementary Figure S2. The differences in species composition among the three groups of samples. A: 6%±M17 27-6; B: 6%; C: 15%

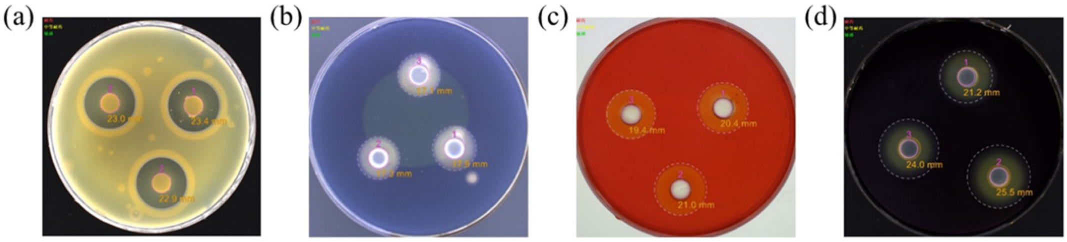

Supplementary Figure S3. Extracellular enzyme hydrolysis circle diagram of *P. polymyxa* M17 27-6.
